# Cognitive Impairment in Heart Failure: Landscape, Challenges, and Future Directions

**DOI:** 10.3389/fcvm.2021.831734

**Published:** 2022-02-07

**Authors:** Mengxi Yang, Di Sun, Yu Wang, Mengwen Yan, Jingang Zheng, Jingyi Ren

**Affiliations:** ^1^Heart Failure Center, China-Japan Friendship Hospital, Beijing, China; ^2^Department of Cardiology, China-Japan Friendship Hospital, Beijing, China; ^3^Department of Neurology, China-Japan Friendship Hospital, Beijing, China; ^4^Vascular Health Research Center of Peking University Health Science Center, Beijing, China

**Keywords:** cognitive impairment, heart failure, epidemiology, pathophysiology, diagnosis, management

## Abstract

Heart failure (HF) is a major global healthcare problem accounting for substantial deterioration of prognosis. As a complex clinical syndrome, HF often coexists with multi-comorbidities of which cognitive impairment (CI) is particularly important. CI is increasing in prevalence among patients with HF and is present in around 40%, even up to 60%, of elderly patients with HF. As a potent and independent prognostic factor, CI significantly increases the hospitalization and mortality and decreases quality of life in patients with HF. There has been a growing awareness of the complex bidirectional interaction between HF and CI as it shares a number of common pathophysiological pathways including reduced cerebral blood flow, inflammation, and neurohumoral activations. Research that focus on the precise mechanism for CI in HF is still ever insufficient. As the tremendous adverse consequences of CI in HF, effective early diagnosis of CI in HF and interventions for these patients may halt disease progression and improve prognosis. The current clinical guidelines in HF have begun to emphasize the importance of CI. However, nearly half of CI in HF is underdiagnosed, and few recommendations are available to guide clinicians about how to approach CI in patients with HF. This review aims to synthesize knowledge about the link between HF and cognitive dysfunction, issues pertaining to screening, diagnosis and management of CI in patients with HF, and emerging therapies for prevention. Based on data from current studies, critical gaps in knowledge of CI in HF are identified, and future research directions to guide the field forward are proposed.

## Introduction

Both heart failure (HF) and cognitive impairment (CI) are the important health concerns for older adults and loom as the public health problems in the coming decades due to the aging global population (1, 2). The similar epidemiological trends and the bidirectional feedback interactions between the heart and the brain are expected to cause a major increase in the prevalence of CI in HF (3). HF is generally considered a leading cause of hospitalization and mortality with an estimated prevalence of >64 million individuals worldwide (4, 5). CI is a very frequent comorbidity in patients with HF and is increasingly recognized as the major cause of chronic disability. It thus confers a substantial global burden to patients and healthcare systems.

Cognitive function refers to a group of mental processes containing memory, language, executive function, visuospatial, concentration, and social cognition (6). The definition of CI is a clinical syndrome that acquires objective cognitive dysfunction affecting one or more cognitive domains. Using normative neuropsychological criteria, CI mostly refers to a performance 1.5 SD units lower than the population mean after accounting for demographics such as age and education. According to the impairment of activities of daily life, CI is classified into mild CI (MCI) and dementia (7). MCI is a stage between normal cognition and dementia that individuals, particularly, those with objective CI on neurocognitive testing and with largely preserved activities of daily living, have. Conversely, dementia is severe enough to affect independent activities of daily life (8).

Cognitive impairment (CI), including its extreme form dementia, has tremendous consequences not only for reduced HF self-care and independence, but also in limiting recognition and appropriate response to worsening HF symptoms of patients. This consequently deteriorates the prognosis of HF (9). It has been confirmed that HF contributes to cognitive decline and that the grade of CI correlates with the severity of HF. As a potent and independent prognostic factor, CI significantly increases the mortality in patients with HF (10). However, some degree of cognitive decline is typical in normal aging; hence, nearly half of CI in HF may be underdiagnosed (11). Therefore, identified CI, especially the early diagnosis of MCI in the preclinical stage, may avoid the occurrence of dementia and, by optimal therapies, revert cognition to normal which has been considered as a crucial strategy for the improvement of prognosis and quality of life in HF population.

In this review, we synthesize knowledge about the landscape of CI in HF, the latest epidemiological data on CI in HF, the current understanding of heart and brain interaction, and the clinical diagnosis and assessment of CI in HF. We also address the potential therapeutic opportunities for preventing and halting the progression of CI in patients with HF. Based on data from current studies, we identify the critical gaps in knowledge of CI in HF and propose future research directions to guide the field forward.

## Epidemiology, Prevalence, and Prognostic Implications of CI in HF

### Epidemiology of CI in the General Population

**Figure 1:**
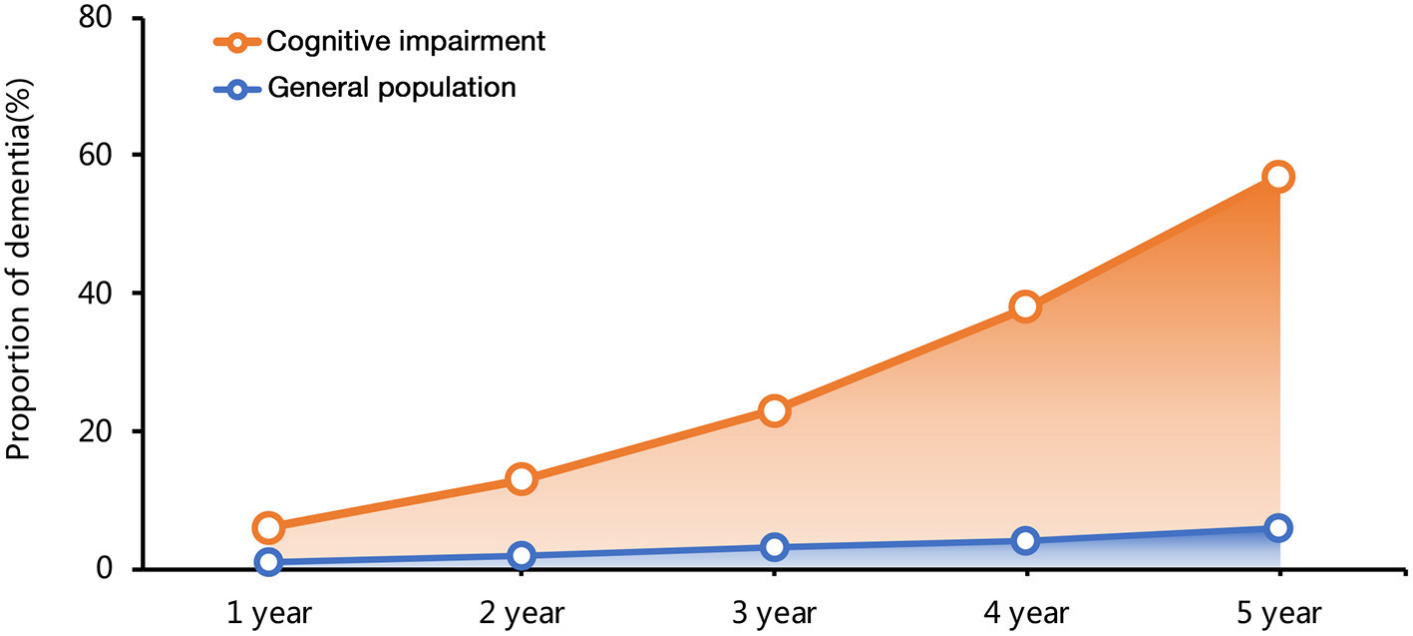
The time curve of dementia incidence in general population and patients with cognitive impairment (CI).

Cognitive impairment (CI) remains a rising global epidemic, accounting for substantial morbidity. The prevalence rates of CI exponentially increase with increasing age, ranging from about 20% to more than 40% in general older adults (12–15). Dementia, the most severe state of CI, affects about 55 million of the adult population worldwide. The incidence of dementia in the general population is 1–2% per year. However, the incidence among patients with MCI that progressed to dementia is significantly higher, with an annual rate of 5–12%, in community-based populations without intervention (14, 16, 17) (Figure 1). The annual death rate was 8% among those with CI, and the rate almost doubled if patients combined with additional medical conditions, such as heart disease (14).

### Prevalence of CI in Patients With HF

**Figure 2:**
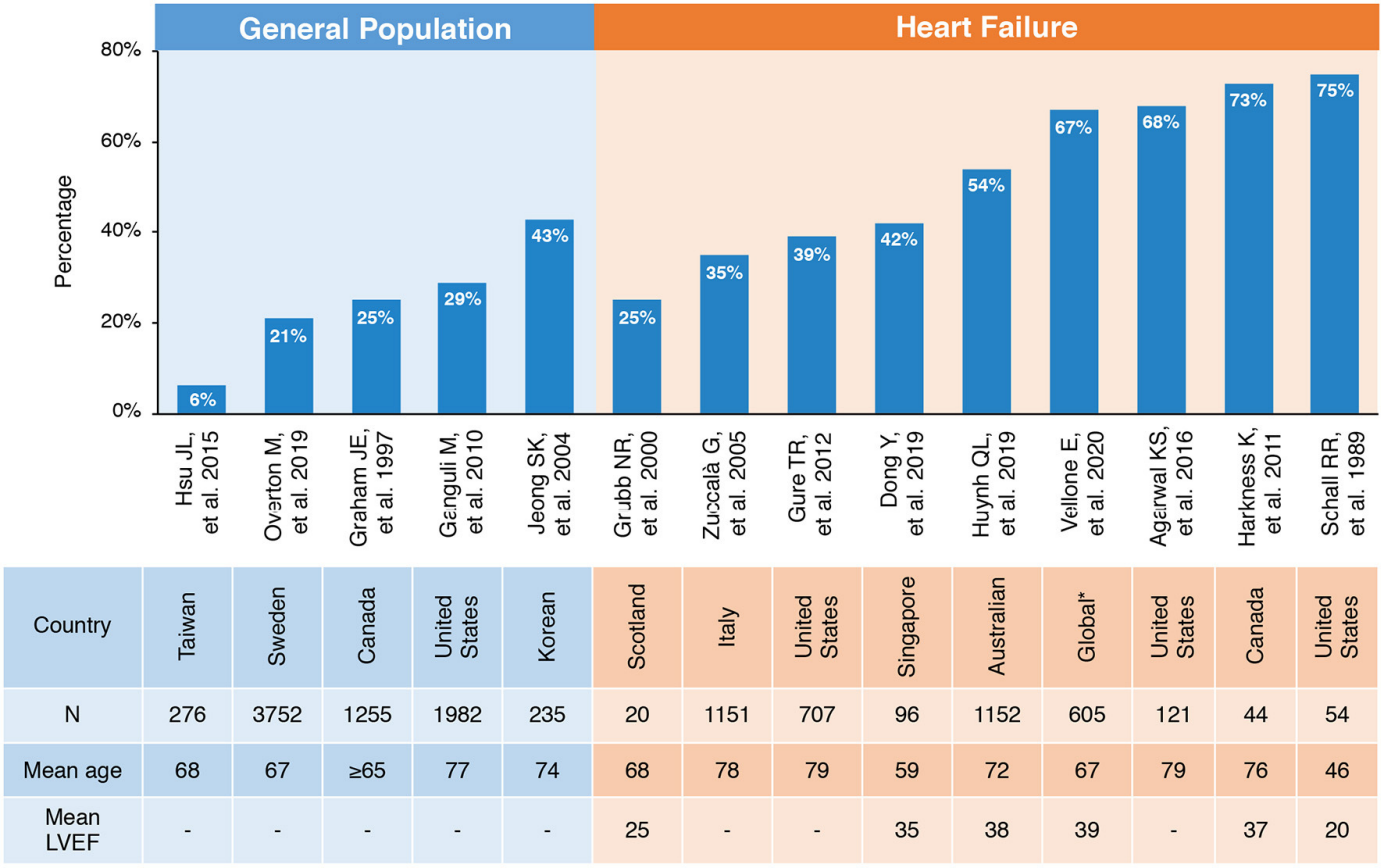
Prevalence of CI in general population and patients with HF. *Including Sweden, Italy, Israel, The Netherlands, Germany, and the United States. CI, cognitive impairment; HF, heart failure; LVEF, left ventricular ejection fraction.

Cognitive impairment (CI) is overwhelmingly common and has become a severe burden in HF with a prevalence of 25–75% across population-based studies due to variation in definitions and diagnostic criteria (Figure 2) (10, 11, 18–23). Accounting for age, the prevalence of CI in patients with HF is significantly higher compared with the general population. A multicenter survey conducted in Italy reported that about 35% (526/1,511) of patients with HF were detected with CI, while only 29% (3,448/11,790) of patients without HF were diagnosed with CI (10). A cross-sectional analysis from communities in US recruited 6,189 patients aged ≥ 67 years. It also found that the age-adjusted prevalence of CI is about 40% in 707 patients with a moderate or high probability of HF, of which more than one third were dementia. The odds of dementia in those with HF were 1.52-fold than that of non-HF patients with the adjustment of age, race, educational level, net worth, and self-reported prior stroke (23). Gallagher R et al. studied 128 HF patients with an average age of 80 years. The odds of CI were increased more than 4-fold in HF caused by ischemic heart disease compared with non-ischemic HF (OR, 4.18; 95% confidence interval, 1.15–15.69) (24, 25). With an exception for chronic HF, the Rehabilitation Therapy for Older Acute Heart Failure Patients (REHAB-HF) study revealed that 78% elderly patients hospitalized with acute decompensated HF had broad marked impairments in cognitive function. In addition, the prevalence of CI was similar in patients with preserved vs. reduced ejection fraction [EF; HF with preserved ejection fraction (HFpEF) and HF with reduced ejection fraction (HFrEF)] when adjusted for sex, body mass index (BMI), and comorbidities (26).

Furthermore, the occurrence and deterioration of CI in patients with HF has been demonstrated in longitudinal studies. Cardiovascular Health Study (CHS) enrolled 4,864 participants without a history of HF and of clinical stroke. After a diagnosis of HF at 80 years old, 5-year decline of cognitive functions was significantly worse compared with that in participants without HF of the same age period (27). Longitudinal data with a longer 8-year period of evaluating trajectories of 457 patients also demonstrated that congestive HF predicted cognitive decline (28).

### Multiple Cognitive Domain Impairments in HF

Recent studies have paid further attention to the detailed and multiple cognitive domains and spectrum of brain lesions in patients with HF. It is well-acknowledged that patients with HF typically exhibit CI in domains of memory, particularly in both verbal and visual memory, working memory, attention, processing speed, and executive function (29). A secondary analysis of Atherosclerosis Risk in Communities (ARIC) study supported that the risk of developing CI had no concern with HFpEF or HFrEF, while worse diastolic function was weakly but significantly associated with worse performance in memory, attention, and language due to abnormal cardiac hemodynamics (30). Similarly, Anna Frey et al. enrolled 148 patients with HF and determined that patients with HF exhibited cognitive deficits in the domains of attention and memory with a prevalence of 41 and 46%, respectively. Furthermore, the degree of advanced medial temporal lobe atrophy (MTA) was strongly related to CI (31). Ichijo et al. assessed the frontal brain activity by near-infrared spectroscopy (NIRS) and non-invasively measured regional cerebral bold volume in patients with HF, showing that frontal brain activity was significantly lower in the HF group than in the control subjects (28.5 vs. 88.0 mM mm; *p* < 0.001) and significantly correlated with mini-mental state examination (MMSE) (*R* = 0.414, *p* = 0.017) (32). Similar data from Asian populations indicated that the neuropsychological impairment in Asian patients with HF characterized vascular pathology with frequently impaired visuomotor speed (60%), visuoconstruction (48%), and visual memory (43%) (11).

### Deterioration of Prognosis With CI in HF

Heart failure (HF) and CI accelerate each other, and CI would further worsen the cardiac function and prognosis of HF with higher mortality, hospitalization admission, and poor quality of life (QoL). As early as before the incident HF, CI is prevalent in patients with subclinical chronic heart disease at high-risk of chronic HF. A prior study supported that patients with MCI had 2-times higher risk with diastolic dysfunction and 1.7-times greater risk with other cardiac abnormalities (33). Moreover, the development of CI in patients with HF has an adverse impact on the clinical outcomes. Previous studies confirmed that CI was an independent risk factor for death and readmission in patients with HF, which increased the risk of cardiovascular mortality by 57% and the risk of all-cause death by 50%. The prospective multi-center prevalence and prognostic value of social frailty in geriatric patients hospitalized for HF (FRAGILE-HF) study enrolled 1,180 patients with HF aged ≥65 years, and 37.1% were identified as with CI using Mini-Cog. Further, they observed that coexistence of multiple frailty domains, including cognitive dysfunction, was prevalent in patients with HF readmission and all-cause death within 1 year (34). Similarly, Patel A et al. recruited 270 patients with HF and reported that the all-cause death and readmission rates of patients with CI were twice as high as those of patients without CI (46 vs. 22%, *p* < 0.0001) (35). In line with them, Hannes H et al. further recognized that worse scores of Montreal Cognitive Assessment (MoCA) heralded increased mortality and readmission risk. A study screened HF patients aged ≥70 and found that patients with HF with CI had a significantly higher 30-day readmission rate than those without CI (26.8 vs. 12.8%; *p* < 0.05) (36). CHS study examined CI and other common comorbidities in 558 participants who developed incident HF and showed that CI was significantly associated with greater total mortality risk (37). Additional investigations regarding the cognitive functions were conducted and worse outcome were observed. Huynh QL et al. performed MoCA in 1,152 Australian patients with HF with a 12-months follow-up, suggesting that visuospatial/executive and orientation were the cognitive domains that were most predictive of post-discharge adverse outcomes in HF (18). It should also be noted that even MCI could deteriorate the prognosis of patients with HF.

It is widely endorsed that patients with HF are vulnerable to CI with serious consequences in healthcare and outcome, with interplay of poor self-care, incapacity of adhering to treatment regimens, and weakening daily living due to decreased attentions, memory, and execution abilities (38–40). On the other hand, the difficulties in describing symptoms would interfere with the recognition by doctors of worsening HF, resulting in the inability to adjust treatment strategy in time. Apart from the adverse outcome brought from poor disease management, CI also seriously impairs QoL and exercise capacity of patients. The secondary analysis of the Wii-HF trial, conducted with 605 patients, evaluated CI using MoCA and measured exercise capacity using a 6-min walk test (6MWT). It indicated that lower 6MWT scores were associated with five domains in cognitive function, including visuospatial/executive, naming, attention, language, and orientation (19).

## Pathophysiology, Cellular Mechanism, and Risk Factors of CI in HF

**Figure 3:**
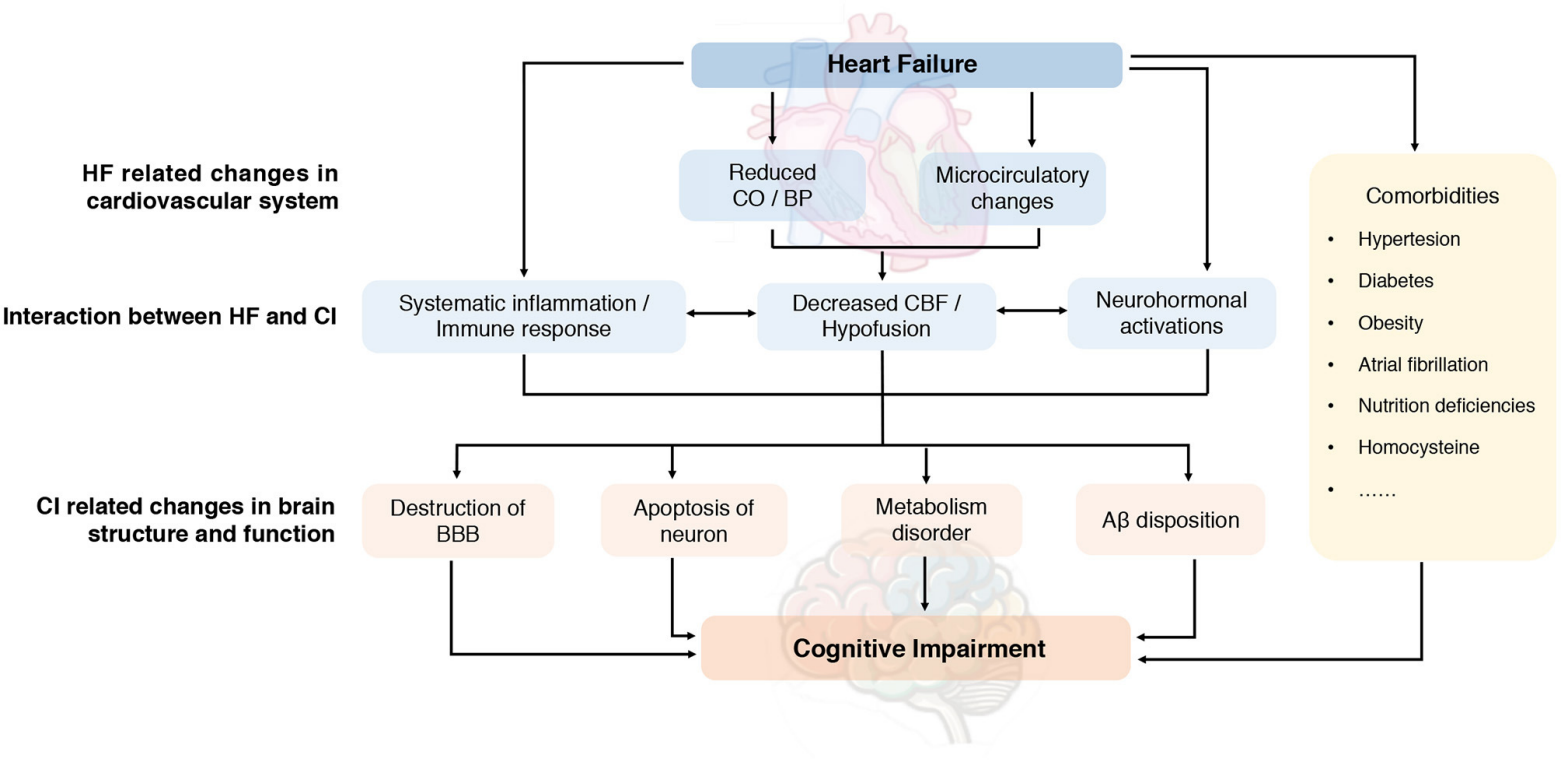
Proposed pathophysiology of cognitive impairment in heart failure. Aβ, amyloid-β; BBB, blood-brain barrier; BP, blood pressure; CBF, cerebral blood flow; CI, cognitive impairment; CO, cardiac output; HF, heart failure.

The underlying mechanism proposed for CI in HF is multifactorial but not fully elucidated. It is important to understand the pathophysiological mechanism regarding CI in HF for a promising diagnostic and therapeutic approach. Existing evidence indicated that hemodynamic alterations and molecular mechanisms may play important roles in the interaction between HF and CI. Also, common cardiovascular and non-cardiovascular comorbidities burden further link HF and CI with risk factors which have not been well established. A systematic summary addressing the potential pathophysiology and mechanisms is illustrated in Figure 3.

### Pathophysiology of CI in HF

#### Reduced Cerebral Blood Flow

It has well-established that HF usually has unfavorable effect on cerebral perfusion with decreased cerebral blood flow (CBF) (41). According to previous studies, CBF could be reduced by nearly 14–30% in chronic HF depending on the severity and chronicity of HF. Despite this, it can be managed using medical treatment, cardiac resynchronization therapy, left ventricular assist devices and heart transplantation (42–46). Several factors determine the reduced CBF in patients with HF. In addition to the systematic hypoperfusion caused by reduced cardiac output (CO) and blood pressure (BP), the distortion of cerebrovascular autoregulation also appear to play critical roles in decreasing CBF (47). The cerebral autoregulation ability help maintain the adequate blood flow in the brain after using vasodilators. However, in patients with HF, microcirculatory changes, such as endothelial dysfunctions, reduced nitric oxide bioavailability, and vascular smooth muscle proliferations may lead to impaired cerebral autoregulation and abnormal cerebrovascular reactivity which appears as significant determinants of reduced CBF in HF (48).

Due to high metabolic demand and limited capacity for energy stores, the normal functions of brain highly depend on adequate perfusion to maintain normal nerve activity. Thus, the subtler alterations of CBF impairment and hypoperfusion due to cardiac dysfunctions could cause chronic brain injury, especially the vulnerable areas in the brain (49). A recent study further calculated the whole-brain CBF maps using MRI scanner and found that reduced CBF appeared in multiple areas involving bilateral prefrontal, frontal, temporal and occipital cortex, thalamus, cerebellum, corona radiate, corpus callosum, hippocampus, and amygdala, which regulate memory, decision-making executive functions, and language (50). Additionally, prior data has revealed that white matter lesions (WML) were expanded in patients with HF and was associated to CI (51, 52). This data is regarded as a manifestation of cerebral small vessels. The extensively decreased cerebral perfusion in cognitive regulatory sites is likely to contribute to cognitive deficits.

The deleterious effects of local CBF loss on cognitive function regarding molecular level have not been fully clarified, while hypoxia, reduced metabolic activity, and neurohormonal activations may play crucial roles to contribute to brain injury (53). Hypoxia caused by hypoperfusion induce the release of hypoxia inducible factor-1 (HIF-1) and increase the expression of vascular endothelial growth factor-1 (VEGF-1), resulting in increased permeability and disruption of the blood-brain barrier (BBB) by destroying the tight junctions (54, 55). Moreover, it has been shown that the reduced CBF and chronic perfusion could decrease the ability of glial cells to eliminate amyloid-β (Aβ).

Even jeopardized CBF has commonly been considered to be the key explanations for CI in patients with HF as data on CBF in HFpEF with the similar manifestation of CI are still insufficient (56). Additionally, in the case of cortical gray matter loss with rich vasculature, reduced CBF did not seem to be the main causative factor (57), implying more pathological offenders should be investigated.

#### Inflammation, Oxidation, and Immunity

Amounting studies have shown that HF is considered as a state of systematic inflammation and that hypoperfusion in HF could contribute to local brain inflammation, which may also paly critical roles in development and deterioration of HF associated CI. Oxidative stress usually interplays with inflammation and is also proposed as one of the mechanisms in cognitive decline (58). Inflammatory factors, including interleukin-1 β (IL-1β), interleukin-6 (IL-6), tumor necrosis factor-α (TNF-α), CRP, and interleukin-17A (IL-17) have been found to be inversely associated with cognitive functions in HF patients (59) as these remain significant after changes in LVEF and symptoms of HF. Additionally, animal models of HF also demonstrated that the expression of inflammatory genes, such as toll-like receptor-4 (TLR-4), TNF-α, and IL-6, were significantly upregulated in the cortex and hippocampus, particularly in the mouse (60). Cytokines regulate cognition by altering the synaptic plasticity and neurogenesis and directly inhibit the neurotransmitter cascade involved in learning and memory (61, 62). Among the proinflammatory factors, IL-1β and TNF-α seems to be the main driver and regulator in the inflammatory response, causing cell death *via* increasing neurotoxicity. IL-1β decreases the release of glutamate, which further affects the release of brain-derived neural factors and promotes the activation of protein kinase by p38 mitogen in the hippocampus, thus interfering with the memory and consolidation ability of hippocampus (63). ASK1-p38-TNF-α is one of the key pathways involved in the disruption of the BBB, inducing the production of IL-6 (64). At the same time, IL-1β and TNF-α would upregulate the expression of CD73, which exert a protective effect on WHL by activation of glial cells and help to relieve inflammation *via* the counter-regulatory feedback (65).

Oxidative stress and immune response usually act synergistically. Increased level of circulating angiotensin II (Ang-II) during HF process induced perivascular macrophage (PVM) activation, which further regulate vascular permeability and recruit granulocyte. Upon Ang-II binds to angiotensin receptor 1 in the PVMs, the NADPH oxidase 2 is activated to promote ROS overproduction (66, 67). ROS, such as superoxide, NO, and ONOO–, increased BBB permeability *via* activating matrix metalloproteinase, producing oxidative damage to cellular molecules (68). Oxidative damage could also activate β-site amyloid precursor protein-cleaving enzyme-1 (BACE-1), resulting in increased synthesis of amyloid precursor protein (APP) and Aβ (60, 69). Meanwhile, the abnormality downregulated expressions of β-site amyloid precursor protein-cleaving enzyme-2 (BACE-2) and contribute to reduced degradation of Aβ precursor proteins.

During the process of Aβ synthesis and deposition, evidence suggest that immune mediator, such as microglia and the macrophages in central nervous system, may contribute to the disease progression (70). Altered phenotype of microglia due to hypoperfusion result in impaired functions, which further reduce the elimination of Aβ (71). The high level of signal of translocator protein (TSPO) often indicate hyperactivated microglia. Thus, TSPO has been found as a useful marker to identify microglial activity. At the same time, active astrocytes express more amyloid precursor protein and cause an increasing Aβ production (72). Dendritic spines are important in learning and memory functions. When they are destroyed by excessive neurons, death, or Aβ, CI would occur (73).

In brief, the inflammation, oxidative stress, and immune response aggravate cognitive functions mainly *via* disruption of the BBB, damaging white matter and actitation of glial cells and, consequently, leading to CI (74).

#### Neurohumoral Activations

Although it has been well-documented that reduced CO and CBF are major drivers for CI in HF, interestingly, HFpEF without diminished brain perfusion has also proven to be closely and independently associated with CI. Therefore, current data support the notion that CI in HFpEF may be associated with additional mechanisms independent of hypoperfusion, of which neurohumoral activations have caught increasing attractions (75). Exacerbated neurohumoral activations may alter neuronal functions and promote productions of CI-related proteins in cognitive areas.

Sympathetic nervous system and renin-angiotensin system (RAS) activation and elevated catecholamine levels in HF were related to poor cognitive performance. On one hand, the exaggerated sympathetic activity and RAS participate in the rightward shift of the lower limit of CBF autoregulation and lead to CBF reduction in patients with HF (45). On the other hand, it is speculated that increased level of catecholamines in HF could disturb Wnt signaling *via* inducing a loss of β-adrenergic pathway. Wnt/β-catenin signaling has been implicated in the regulation of synaptic assembly, neurotransmission, and synaptic plasticity of hippocampus. Toledo et al. found that a rat with HF with normal EF displayed impaired learning process and memory loss. CI in the rats was correlated to the downregulated Wnt/β-catenin signaling, which attenuated phosphorylated glycogen synthase kinase 3β (p-GSK3β) in the hippocampus and reduced synaptic plasticity, ultimately impairing cognitive functions (76, 77).

In addition, sustaining activation of the neuroendocrine system, such as hypothalamic-pituitary-adrenal axis, was suggested to have potential effects on progression of HF and structural damages of brain by regulating the neuronal metabolism, physiologies, and gene expressions. Glucocorticoid receptors are commonly expressed in neurons and glial cells with the highest levels in the hypothalamus, hippocampus, and amygdala brain structures, while the mineralocorticoid receptors expressed most in the hippocampus, amygdala, and prefrontal cortex edge. The two kinds of cortisol receptors play vital roles for the normal cognitive functions (78).

### Potential Cellular Mechanisms of CI in HF

#### Disruption of Blood-Brain Barrier

The complete BBB exert protective effects by preventing extravasation of toxic substances from circulation to the brain parenchyma. The increased permeability of BBB mainly caused by hypoperfusion and inflammation would lead to the influx of fluids, ions, albumin, and other proteins into neurons from the blood and cause infiltration of immune cells and secondary inflammation, further exacerbating brain edema, oxidative damage, luminal stenosis, and neuronal dysfunction, and ultimately lead to CI in patients with HF (79, 80).

#### Metabolism Disorder

The normal energy metabolisms in the brain are high reliant on proper cardiac function, thus poor perfusion and ischemia lead to rapid consumption of adenosine triphosphate (ATP) and subsequent ROS are produced. Oxidative damage further increases the production of cytokines to induce specific inflammatory changes and lead to Aβ deposition eventually (81). In addition, mitochondrial dysfunction has been suggested as a key mechanism during development of CI in HF, as energy deficiency would result in functional abnormalities of central neurons which are intimately linked to cognitive functions (82). Incremental evidence indicated that mitochondria are also critical for neurodevelopment and neurogenesis, while mitochondrial degeneration could mediate CI through Wnt signaling pathway (83).

#### Apoptosis of Neuron

Loss of brain cells has been observed in lots of neurological diseases. Bax, a member of the Bcl-2 family, plays a key role in regulating apoptosis. Prior studies have found that the expressions of Bax in the hippocampal cortex changed female mice with HF, which may affect the apoptosis process of neurons related to cognitive function in the brain (84). Furthermore, evidence have shown that the expression of caspase family, especially caspase 3 and 6, have been increased in the hippocampal tissue of mice with HF (85). Animal studies also found that the activation of the RAS in mice with HF can affect AMPK-PGC1α signaling by stimulating angiotensin II receptors and increase the apoptosis of neural stem cells in the hippocampus of rats (86).

#### Amyloid-β Deposition

Neuropathology of Alzheimer's disease (AD) such as Aβ deposition is closely related to chronic hypoperfusion. In patients with HF, a variety of pathways may participate in the accumulation and deposition of Aβ *via* increasing production and decreasing clearance, including CBF insufficiency, activation of microglia and astrocytes, inflammatory cascade, and oxidative imbalance. In turn, Aβ could aggravate these pathological alterations and promote neurodegenerative process, forming a vicious circle. The accumulation of Aβ ultimately leads to CI in patients with HF (87).

### Risk Factors Predicting CI in Patients With HF

Although it is well-known that patients with HF are more prone to cognitive decline, the predictors of developing CI have not been fully clarified. Some earlier studies and systematic reviews revealed that left ventricular ejection fraction (LVEF) and 6-min walk tests (6MWT) were independently associated with development of CI in patients with HF. Recent work also pointed out that the high level of NT-proBNP was the independently predictor for CI in patients with HF (11, 88–90). In addition, the risk factors for dementia-related structural brain damage have also been explored. Karsten et al. observed a significant correlation between diminished gray matter density (GMD), decreased LVEF, increased NT-proBNP, and GMD in wide brain regions including the whole front median cortex along with the hippocampus and precuneus (91).

To date, the role of the well-established comorbidities in CI, such as diabetes mellitus (DM) and hypertension, has been controversial in HF-associated CI. The longitudinal time-varying analysis of Warfarin vs. Aspirin in Reduced Ejection Fraction (WARCEF) trial disclosed that higher baseline cognitive status (MMSE scores), non-white race, older age, lower education, and NYHA ≥ II were independently associated with cognitive decline in HF while traditional cardiovascular risk factors containing hypertension, DM, and smoking seemed no association with cognitive decline (92).

Besides, additional comorbidities coexisting with HF contributed to CI have become gradually recognized by exerting inflammatory, metabolic, and neurohormonal pathways (31). Obesity, as a significant contributor to HF and, especially, HFpEF, has become an established risk factor for adverse brain changes and poor cognitive outcome in HF (93). Atrial fibrillation (AF) was also demonstrated to be significantly associated with CI independent of a history of stroke, exhibiting lower total brain, gray and white matter volumes related to poorer cognition, and increased risk of dementia (94, 95). The potential mechanism includes micro-emboli and hypoperfusion resulting from abnormal heart rate and CO. Moreover, nutritional deficiencies are common in patients with HF due to absorption disorders of nutrition or diuretic use. Low level of folate, B12 vitamin, and albumin were correlated with CI and anemia (96). Animal models have demonstrated that thiamine deficiency caused brain atrophy and white matter changes, further affecting the learning ability in rats (3, 97). In addition, homocysteine levels due to renal insufficiency led to brain atrophy and brain cell apoptosis, further affecting neurogenesis and resulting in cognitive deficit (98).

## Clinical Diagnosis and Assessment for CI in HF

### Subjective Assessment

Assessments, including subjective and objective, are crucial for the diagnosis of CI in HF. Whenever possible, history should be obtained both from the patient and from a family member, caregiver, or other reliable informant (99, 100). However, many patients with HF and their families accept cognitive decline as part of normal aging, and will declare themselves normal on the grounds that they are no worse than others their age. Therefore, subjective concerns alone are insufficient for diagnosis.

### Neuropsychological Tests

The objective assessment requires to accomplish one or more standardized neuropsychological tests. Neuropsychological assessment of specific cognitive domains is preferred for both detecting mild impairments and for differential diagnosis. Diagnostic for CI in patients with HF are the same as in populations without HF. Previous studies have shown that the identification of CI by the screening tools was more accurate than the diagnosis by symptoms alone for patients with HF (101). More importantly, early diagnosis and therapy of CI could significantly reduce the 6-month readmission rate and mortality in patients with HF (101).

Multiple neuropsychological tests are available for CI assessment, including the MMSE (102–106), the MoCA (103, 105, 107), the Mini-Cog (108, 109), the Saint Louis University mental status (SLUMS) (110), the rapid cognitive screen (RCS) (111), and the Cambridge cognitive examination (CAMCOG) (112–114). MMSE and MoCA are the most frequently used tests in clinical practices. Due to different cut points, the sensitivity and specificity of screening tools are different (Table 1). It is critically important that the test performance of a patient be interpreted in accordance with norms for the age, educational level of that patient, and preferably for his/her cultural/linguistic group and region as well.

**Table 1:** Screening tools used commonly in clinical practice for cognitive impairment (CI) diagnosis and assessment in general population.

**Classification**	**Screening**	Cut points^*^	**Sensitivity (%)**	**Specificity**
	**tools**			**(%)**
MCI	MMSE (103)	≤ 22~29	62~85.5	53.0~65.9
	MoCA (103)	≤ 22~27	68.7~93.0	63.9~100.0
	Mini-Cog (109)	≤ 2	55	83
	CAMCOG (113)	≤ 94	72	76
	RCS (111)	≤ 7	87	70
	SLUMS (110)	≤ 23.5~25.5	92~100	55~81
Dementia	MMSE (104–106)	≤ 23~26	87~89	82~89
	MoCA (105)	≤ 17~23	93	90
	Mini-Cog (106)	≤ 2	76~99	89~96
	CAMCOG (114)	≤ 92~93	100	95
	RCS (111)	≤ 5	89	94
	SLUMS (110)	≤ 19.5~21.5	100	91~98

* *The cut points for diagnosis of mild CI (MCI) are dependent on the population norms, age, educational level, and comorbidities, estimates of premorbid cognitive function.*

*MMSE, mini-mental state examination; MoCA, montreal cognitive assessment; SLUMS, Saint Louis University mental status; RCS, rapid cognitive screen; CAMCOG, Cambridge cognitive examination*.

Nowadays, the diagnostic accuracy of neuropsychological tests to screen for CI in populations with HF varies widely in studies. In a study to evaluate the usefulness of MoCA and MMSE compared with the golden standard European Consortium Criteria for diagnosing MCI in HF population, the sensitivity and specificity of MoCA were 82 and 91% and MMSE were 9 and 91% (115). Hawkins et al. also compared the ability of the MMSE and MoCA to detect CI in patients with HF (116). Both tests are useful in identifying the majority of patients with and without CI with the sensitivity and specificity around 60–70%. Other tests, such as the Mini-Cog and the CAMCOG, had a moderate accuracy in populations with HF (117, 118). With up and coming research investigating CI in HF, however, there is still no clear consensus regarding the optimal screening tool for the assessment of CI in HF. The evidence on how and when best to screen cognitive in patients with HF is needed.

### Biomarkers

The neuropsychological tests only have a moderate accuracy for the assessment of CI, thus there is a large need of special biomarkers to support the clinical diagnosis. Cerebrospinal fluid (CSF) biomarkers that reflect the pathophysiology of CI have been increasingly used and are the most common test in neurology to identify CI. Circulating biomarkers would be preferable, as blood is more accessible than CSF. However, only a fraction of brain proteins enters the circulatory system due to the BBB. Of note, considering that patients with HF and CI have multiple pathologies, a broader panel of biomarkers reflecting neuro injury, inflammation, and oxidative stress would be needed.

#### Cerebrospinal Fluid Biomarkers

The exploration of biomarkers in CSF has focused on the core molecules of CI pathogenesis, including Aβ and tau proteins (119, 120). Numerous studies demonstrated that a marked decrease of Aβ42 in CSF can predict and identify CI due to cortical amyloid deposition in the brain (121, 122). In patients with AD, the degree of increase in CSF total tau is around 300% of control levels (123). A meta-analysis of 51 studies suggested that p-tau is also a satisfactory prognostic biomarker for progression of MCI (124).

However, the lumbar puncture for CSF testing is a rare to be accepted in patients with HF, although it is safe and cheap. Recent research investigated the associations of LVEF with CSF biomarkers in older adults (125). Results showed that participants with lower LVEF had higher levels of CSF t-tau and t-tau/Aβ42 ratios. There are few research focusing on the CSF biomarker in HF population. The biomarkers testing is still highly dependent on blood samples which are relatively convenient and suitable in clinical practice.

#### Circulating Biomarkers

##### Amyloid-β in Plasma

Amyloid-β 42 (Aβ42) is the most extensively studied blood biomarker for the diagnosis of symptomatic and prodromal AD and CI. Several studies reported plasma Aβ as a potentially useful biomarkers for the early diagnosis of cognitive dysfunction and for the prediction of its progression in Parkinson's disease and amnestic MCI (126, 127). However, Bayes-Genis et al. measured circulating Aβ40 in 939 consecutive patients with HF and found that there were no differences in circulating Aβ40 levels in HF patients with and without CI at baseline or during follow-up over a median of 4 years (128). Interestingly, in multivariable analysis, including relevant clinical predictors and N-terminal pro-brain natriuretic peptide (NT-proBNP), Aβ40 remained significantly associated with all-cause (HR, 1.22; 95%CI, 1.10–1.35; *p* < 0.001) and cardiovascular death (HR, 1.18; 95%CI, 1.03–1.36; *p* = 0.02), but not with HF-related death (HR, 1.13; 95%CI, 0.93–1.37; *p* = 0.22). Further studies are warranted to identify whether the bloodstream Aβ concentration results in or facilitates Aβ plaque formation in the brain in HF patients.

##### Inflammatory Factors in Plasma

Heart failure (HF) is considered a state of increased inflammatory responses which induce cognitive dysfunction involved in memory loss and impaired executive function. Redwine LS et al. investigated outpatients with HF and found that lower MoCA scores are associated with higher levels of plasma inflammatory biomarkers, such as interferon-γ (IFN-γ), tumor necrosis factor-α (TNF-α), and soluble vascular cell adhesion molecule-1 (sVCAM-1) (129). In an exploratory parallel design study of 69 patients with symptomatic HF, it was observed that changes in C-reactive protein (CRP) and IL-6 levels predicted alterations in MoCA scores (130).

##### Cortisol in Plasma

Cortisol was indicated to influence cognitive function. Elevated serum levels of cortisol were observed in patients with HF compared with healthy controls. More importantly, significantly higher levels of cortisol were found in patients with HF who had symptoms of depression and CI than those free from these symptoms. Besides, matched patients treated with cortisol performed worse on specific cognitive assessments compared with those treated with placebo, suggesting that cortisol levels in HF might influence the development of CI.

##### Natriuretic Peptide in Plasma

Elevated brain natriuretic peptide (BNP) and NT-proBNP are also associated with CI and increased risk for dementia in patients with HF. In recent years, there have been major advances in the development of blood-based biomarkers for CI. However, the sensitivity and specificity of a single circulating biomarker are not significant. Hence, a combination of biomarkers may offer a better diagnosis value for CI in HF.

##### Potential Biomarkers in Blood

Recent developments have also given some novel circulating candidate biomarkers for CI. The novel N-terminal tau fragment (NT1) in plasma is not only a biomarker to distinguish normal, MCI, and AD dementia populations with high specificity and sensitivity, but is also a strong predictor of future cognitive decline and neurodegeneration in healthy elderly individuals (131, 132). A parallel metabolomics analysis in both the brain and blood conducted by Varma et al. identified 26 metabolites from two main classes, sphingolipids and glycerophospholipids, consistently associated with severity and progression of AD (133). Abdullah et al. found that the serum flotillin levels significantly decreased in patients with AD compared with those of non-AD controls, suggesting flotillin, the abundant exosome protein, may be a novel diagnostic marker for AD (134). However, these biomarkers mainly target in AD in the general population. Further studies are needed to validate these findings in patients with CI, especially in patients with HF and CI. Additional technical developments of novel ultrasensitive immunoassay and mass spectrometry methods show promise for blood biomarkers with potential applications as screening tools for CI in HF.

### Neuroimaging

Neuroimaging has contributed to our understanding of the mechanisms by which HF may lead to CI and is recommended by guidelines for CI diagnosis. Structural changes of brain, such as increased white matter hyperintensities, gray matter loss, and brain atrophy, are frequently encountered imaging findings in patients with HF with CI and often preceded by functional changes.

#### Structural Brain Changes in Neuroimaging

##### White Matter

The increase of white matter hyperintensities (WMH) may represent underlying ischemia and impact upon the course of cognitive symptoms, which has been reported as a specific change of HF-related CI. The pathologically characteristics of WMH contains pale myelin sheath, loss of myelin sheath and axon, and mild gliosis. WMH can lead to cognitive decline and increase the risk of depression, anxiety, cerebrovascular events, dementia, and even death. These lesions usually present in small vessel diseases, which are considered to be the result of the destruction of the BBB caused by chronic cerebral hypoperfusion and the subsequent infiltration of plasma into white matter (135, 136). Brain MRI is a sensitive indicator of cerebrovascular disease and could use to image patients with HF with CI as it can reveal a number of asymptomatic findings, including WMH. Beer et al. found that left medial temporal lobe atrophy and deep WMH detected by MRI showed a negative correlation with cognitive scores in patients with HF compared with healthy controls (137). The population-based LIFE-Adult Study quantitated white matter lesions in 2,490 participants and found that the prevalence was independently associated with WML (OR 2.8, 95% CI 1.2–6.5), which was associated with cognitive dysfunction (138). Further, the longer duration of HF independently predicted WML while the duration of hypertension not.

Diffusion tensor imaging (DTI) reflects the integrity of the fiber bundle by detecting the anisotropy and degree of the water molecule diffusion in cerebral white matter fibers, which clearly display the direction and distribution of intracranial white matter fiber bundles after post-product. Kumar et al. found that the axial and radial diffusion was significantly increased and mainly existed in autonomous, analgesic, emotional, and cognitive parts in patients with HF than that in health controls, suggesting that the axon integrity and myelin were injured in patients with HF (139).

##### Gray Matter

The majority of imaging studies in CI have used MRI to investigate changes in gray matter structure. Almeida et al. compared the brain gray matter reduction using MRI and emotional changes between patients with chronic HF and healthy controls (140). It has demonstrated that the prevalence of depression and anxiety was much higher in patients with HF compared with that in healthy controls. Of note, gray matter loss mostly in particular parts of the brain with motional regulation, such as left and right thalamus, left caudate nucleus, left and right posterior cingulate gyrus, left and right parahippocampal gyrus, left upper middle temporal gyrus, and right lower parietal lobe.

##### Brain Atrophy

Studies have shown that half of patients with chronic HF has cortical brain atrophy exists, which is 10-flod higher than that in the control group (31, 91). With the advancement of imaging technologies, the detrimental effects of HF on CI have also been demonstrated with structural alterations of brain. The COGNITION.MATTERS-HF prospective cohort study quantified the concurring dynamics affecting cognitive functions in 148 patients with mild chronic HF and found that cognitive function remained stable with “intensity of attention” as the only domain declining over 3 years (141). Moreover, in patients with HF, the markedly reductions of hippocampal volume observed at baseline was more related to impaired cognitive function, while the total brain volume and the load of white matter change within the limits of normal aging.

#### Functional Brain Changes in Neuroimaging

The use of single photon emission computed tomography (SPECT) enables to detect the distribution of radionuclides in the body. It is widely utilized due to its great value of diagnosis for early metabolic disorder in various diseases. Alves et al. found that the regional cerebral blood flow reductions in patients with HF using SPECT was similar to the regional glucose metabolism disorder in patients with CI by positron emission tomography, which support to a view that congestion dysfunction brain changes of congestion dysfunction may develop in patients with HF, possibly as a consequence of chronic cerebral blood flow hypoperfusion (142). Recently, a prospectively study enrolled 102 patients with HF and 15 healthy controls who underwent gated 99mTc-sestamibi SPECT and found that cerebral metabolism in the whole brain was reduced, especially hippocampus and para-hippocampus areas in patients with HF, while the cerebral metabolism maintained in frontal areas due to its higher sensitivity and self-regulation (143).

Functional MRI (fMRI) is a technique highlighting regional patterns of brain activation based on little change of the deoxy/oxy-hemoglobin ratio (144). Once the neuronal activity is enhanced, the blood flow through the cortex functional region significantly increased, which results a change of deoxy/oxy-hemoglobin ratio. This ratio, due to the different magnetic properties of hemoglobin states, can be measured through MRI and reconstructed in the form of blood oxygenation level–dependent signal. Hence, fMRI has been widely used to describe the characteristics cerebral function and functional networks in CI and its related diseases. Several studies have shown the extensive activity reduction in the brain of patients with diabetes. However, fMRI is rarely used in patients with HF and CI clinically and data specific for HF are currently lacking.

## Prevention And Potential Therapeutic Options for CI in HF

**Figure 4:**
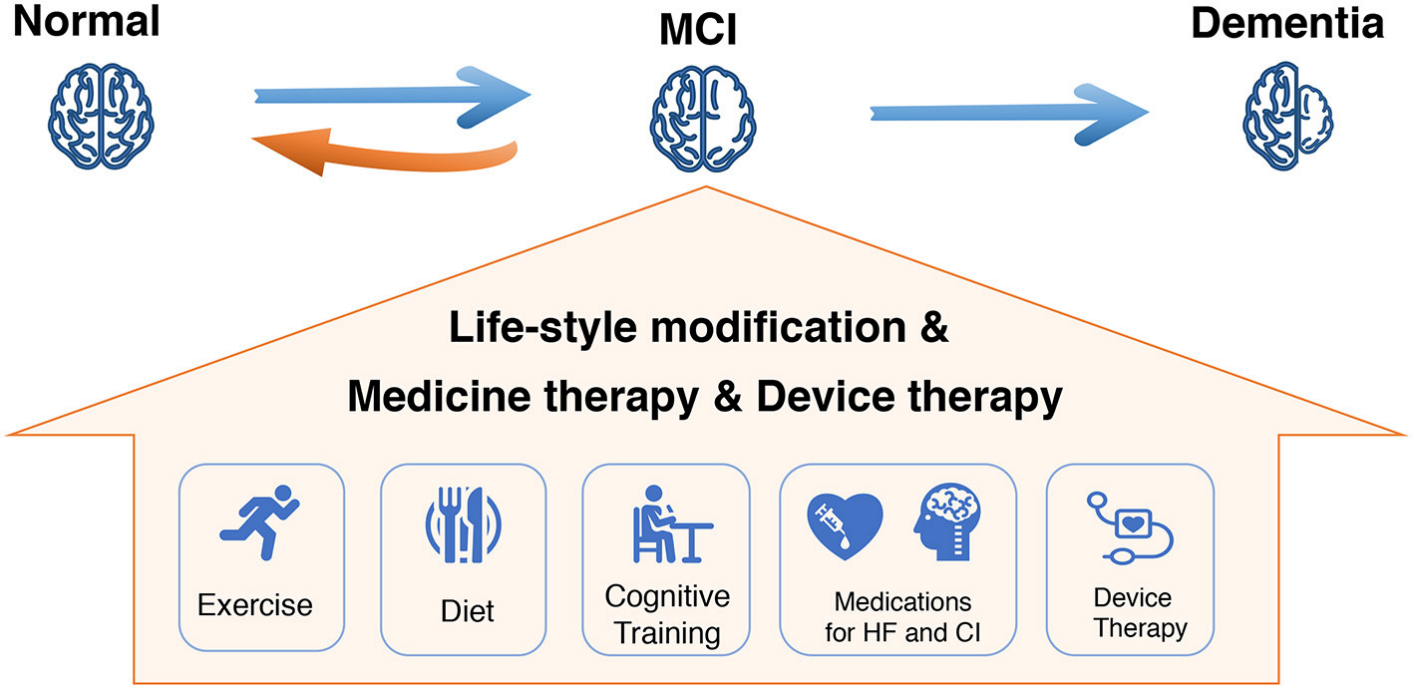
Comprehensive management for mild CI (MCI) in HF to halt disease progression. MCI, mild cognitive impairment; HF, heart failure.

Improving and maintaining cardiac function should be the primary strategy for treatment of CI in patients with HF, which would have a positive impact on brain function (Figure 4). Although clinical guidelines in HF have recently begun to emphasize the importance of CI in HF, few recommendations are available to guide clinicians about how to approach CI in patients with HF. Contemporary HF therapies may improve or aggravate cognitive decline (Table 2). On the other hand, therapy for CI may also have cardiovascular side effects, which may in turn affect the treatment of HF. Furthermore, research are undergoing to develop novel potential therapeutic targets for CI and HF (Table 3).

**Table 2:** Summary of trials targeting medical treatment of cognitive impairment in heart failure (HF) (completed).

**Trial**	**Year of publication**	**Study design**	**Intervention**	**Study population (*n*)**	**Primary end point**	**Secondary end points**	**Results**
**ACEIs/ARBs**							
The antihypertensives and vascular, endothelial and cognitive function trial (AVEC trial) (157)	2012	Randomized, controlled	Candesartan vs. lisinopril vs. hydrochlorothiazide	hypertension and cognitive impairment aged ≥60 years (53)	Changes in cognitive assessment: making test parts A and B (TMT), Hopkins verbal learning test—revised (HVLT), and the digit span test.	Change of systolic blood pressure and blood flow velocity	Candesartan improved in TMT-B (*P* = 0.008), the adjusted TMT, B-A which adjusts the test for motor speed (*P* = 0.012), and the recognition portion of the HVLT (*P* = 0.034). Blood pressure control levels and systolic blood pressure reductions were equivalent in all three groups.
Soto et al. (151)	2013	Randomized, controlled	ACEIs vs. other antihypertensive drugs	Older adults with mild to moderate Alzheimer's disease (616)	Change in MMSE score.	-	The use of ACEIs in older adults with AD is associated with a slowest rate of decline in MMSE score independent of hypertension.
Ginkgo evaluation of memory study (GEMS) (158)	2013	A *post-hoc* analysis of the randomized controlled GEMS trial.	ACEIs vs. diuretic vs. ARB vs. β-blocker vs. CCB	Older adults aged ≥75 years with normal cognition (1,928) or MCI (320)	Incidence of AD.	-	ACEI, ARB, or diuretic use was associated with reduced risk of AD among patients with normal cognition or MCI.
Zuccalà et al. (150)	2005	Observational, retrospective	With ACEIs vs. without ACEIs	Heart failure (1,220)	The improvement of cognitive performance	-	Cognitive performance improved in 30% of participants started ACEIs, but only in 22% of remaining patients (*P* = 0.001). Use of ACEIs among patients with heart failure was associated with improving cognition (odds ratio = 1.57; 95% CI 1.18–2.08) in the multivariable regression modeling, independently of baseline or discharge blood pressure levels. The probability of improving cognitive performance was higher for dosages above the median values compared with lower doses (odds ratios = 1.90 and 1.42; *P* for trend = 0.001), and increased with duration of treatment (odds ratios for the lower, middle, and upper tertiles = 1.25, 1.34, and 1.59; *P* for trend = 0.007)
Ouk et al. (160)	2021	Observational, retrospective	ACEIs vs. ARBs	Cognitively normal or AD/MCI with amyloid-β-positive aged 55–90 years (311)	Global and sub-regional amyloid-β accumulation by ^18^F-flflorbetapir	-	In cognitively normal older adults, ARB use was associated with a lower rate of global Aβ accumulation over time compared to ACE-I users. The rates of amyloid-β accumulation had no difference between ARBs or ACEIs in amyloid-positive participants with AD dementia or MCI.
Hajjar et al. (159)	2020	Randomized, controlled	Candesartan vs. lisinopril	hypertension and mild cognitive impairment aged 55 years or older (141)	Change in executive function (measured using the trail making test, executive abilities: measures and instruments for neurobehavioral evaluation and research tool)	Change in episodic memory (measured using the Hopkins verbal learning test-revised) and microvascular brain injury reflected by MRI of white matter lesions.	Candesartan was superior to lisinopril on the executive function measured by Trail Making Test Part B [effect size (ES) = −12.8 (95% CI, −22.5 to −3.1)] but not executive abilities: measures and instruments for neurobehavioral evaluation and research score. Candesartan was also superior to lisinopril on the secondary outcome of Hopkins Verbal Learning Test-Revised delayed recall [ES = 0.4 (95% CI, 0.02–0.8)] and retention [ES = 5.1 (95% CI, 0.7–9.5)].
**β-blockers**							
Holm et al. (162)	2020	Randomized, controlled	With β-blockers vs. without β-blockers	General population treated with β-blockers (18,063)	Incidence of dementia (developing vascular dementia, all-cause, Alzheimer's and mixed dementia)	-	β-blocker therapy was independently associated with increased risk of developing vascular dementia, regardless of confounding factors (HR: 1.72, 95%CI 1.01–3.78; *P* = 0.048). Conversely, treatment with β-blockers was not associated with increased risk of all-cause, Alzheimer's and mixed dementia
**ARNI**							
PARADIGM-HF. (146)	2017	Randomized, controlled	Sacubitril/valsartan 97/103 mg bid vs. enalapril 10 mg bid in a 1:1 ratio	Symptomatic HFrEF (8,399)	Relevant cognition- and memory-related adverse event (AE) reports	-	The incidence of dementia-related AEs in patients treated with sacubitril/valsartan was similar to that in patients treated with enalapril.
**SGLT2 inhibitor**							
Simone et al. (156)	2018	Randomized, controlled	Incretins vs. SGLT2 inhibitor	Elderly patients with type 2 diabetes mellitus (39)	Change of cognitive performance with the attentive matrices test, the verbal fluency test and the Babcock story recall test.	Metabolic outcomes.	Cognitive status did not change significantly during the 12 months of treatment in SGLT2 inhibitor group or incretins group. SGLT2 inhibitor resulted in a reduction in weight, in BMI, and an increase in high-density lipoprotein cholesterol.
Mui et al. (148)	2021	Retrospective, territory-wide cohort study	SGLT2I vs. DPP4I	Type 2 diabetes mellitus patients (39,828)	New-onset dementia, Alzheimer's, and Parkinson's.	All-cause, cardiovascular, and cerebrovascular mortality.	SGLT2I users had lower incidences of dementia, Alzheimer's, Parkinson's disease, all-cause, cerebrovascular, and cardiovascular mortality. SGLT2I use was associated with lower risks of dementia, Parkinson's, all-cause, cardiovascular, and cerebrovascular mortality.

*Aβ, amyloid-β; ACEIs, angiotensin converting enzyme inhibitors; AD, Alzheimer's disease; ARNI, angiotensin receptor neprilysin inhibitor; ARBs, angiotensin II receptor blockers; MCI, mild CI; MRIs, magnetic resonance images; SGLT2, sodium glucose co-transporter 2*.

**Table 3:** Ongoing trials targeting medical treatment of CI in HF.

**Trial**	**Study design**	**Intervention**	**Study population (*n*)**	**Primary end point**	**Secondary end** **points**	**ClinicalTrials.gov identifier**
**ACEIs/ARBs**						
Candesartan's effects on Alzheimer's disease and related biomarkers (CEDAR)	Randomized, controlled	Candesartan vs. Placebo	Mild cognitive impairment; amyloid positivity determined (77)	Hypotensive episodes, symptoms of hypotension, elevated serum creatinine, hyperkalemia, discontinuing the study medication	Change in cerebrospinal fluid (CSF) Tau levels, pulse wave velocity (PWV), augmentation index (AI), brain perfusion, hippocampal volume, vasoreactivity, clinical dementia rating (CDR)	NCT02646982
**Angiotensin (1–7)**						
Angiotensin (1–7) treatment to improve cognitive functioning in heart failure patients	Randomized, controlled	Drug: angiotensin-(1–07) Behavioral: memory training	Adults with chronic HF; without neurologic or psychiatric disorders (16)	Changes in performance on the Memory Intentions Test (MIST)	Changes of assessment of self-reported quality of life (QoL) and systemic inflammation assay	NCT03159988
**ARNI**						
A multicenter, randomized, double-blind, active-controlled study to evaluate the effects of LCZ696 compared to valsartan on cognitive function in patients with chronic heart failure and preserved ejection fraction	Randomized, controlled	LCZ696 vs. valsartan vs. placebo	Patients with chronic HFpEF and cognitive function (592)	Change in the CogState global cognitive composite score (GCCS)	Change in cortical composite standardized uptake value ratio (SUVr), individual cognitive domains (memory, executive function, and attention), the summary score of the instrumental activities of daily living (IADL)	NCT02884206

### Contemporary Medical Therapies

#### Effect of HF Medical Therapy on CI

##### Sodium Glucose Co-Transporter 2 Inhibitor

The novel antidiabetic agent, sodium glucose co-transporter 2 (SGLT2) inhibitor, is essential for overcoming the burden of diabetic and have beneficial cardiovascular and renal effects, especially in improving the prognosis of HF, which supports it as a foundational therapy for HF (153, 154). Evidence have shown SGLT2 inhibitor to limit or slow down brain pathology in CI among patients with diabetes. Animal studies indicated that SGLT2 significantly ameliorated cognitive decline in type 2 diabetic mice (152, 155). A recent large propensity score-matched population-based study demonstrated that patients treated with SGLT2 inhibitors were associated with lower risks of dementia compared with when treated with DPP4 inhibitors (148). Simone et al. also found that cognitive status did not change significantly during the 12 months of treatment of SGLT2 inhibitor (156). However, whether the SGLT2 inhibitor could reduce incidence of cognitive or improve the cognitive dysfunction in HF is not known and requires further confirmation.

##### Angiotensin Receptor Neprilysin Inhibitor

Angiotensin receptor neprilysin inhibitors (ARNIs) significantly improve the clinical outcome of patients with HFrEF as shown in PARADIGM-HF which compared the angiotensin receptor neprilysin inhibitor (ARNI) (sacubitril/valsartan) with angiotensin converting enzyme inhibitor (ACEI) (enalapril). As neprilysin is one of enzymes clearing Aβ peptides from the brain, in theory, inhibition of neprilysin may reduce Aβ degradation and accelerate its accumulation (145). Besides, inhibition of neprilysin increases bradykinin levels, which directly interacts with Aβ1-42 aggregates to generate Aβ plaques. Hence, there is a concern about how neprilysin inhibitor may cause cognitive decline in patients treated with ARNI. However, large scale randomized controlled trials, such as PARADIGM-HF, confirm that there is no adverse effect of ARNI on CI (146, 147). A possible cause may be that cognitive decline in HF is not wholly related to Alzheimer's type pathology and may also be associated with declining cardiac function and vascular abnormalities. Therefore, the long-term ARNI treatment, by improving cardiovascular function and preventing hospitalization, may have a positive effect on cognitive function in patients with HF. Further, a study, PERSPECTIVE (NCT 02884206), is undergoing to explicitly focused on whether ARNNI will lead to CI in populations with HFpEF. This study is expected to be completed in March 2022.

##### Angiotensin Converting Enzyme Inhibitor/Angiotensin II Receptor Blocker

Angiotensin converting enzyme inhibitors (ACEIs)/angiotensin II receptor blockers (ARBs) have been shown to improve cognitive function in patients with HF by reducing the activity of sympathetic nervous system and improving cerebral blood flow (150). In a mouse model of AD, the centrally active ACEI perindopril significantly reversed the CI, including the indices of immediate working memory and relatively long-term recognition memory (149). In elderly patients with hypertension and CI, studies have indicated that ACEIs and ARBs could improve the cognitive function and were associated with reduced risk of AD (151, 157–159). Recently, research demonstrated that ARBs had a stronger protection against memory decline than ACEIs through its potential benefits on the inhibition of Aβ accumulation in the cortex (160).

##### β-Blockers

Findings of β-blockers to improve CI are heterogeneous, and some are even controversial. β-blockers may reduce the incidence of CI through the control of blood pressure. It has been first reported in *JAMA* in 1986 that β-blockers may lead to depression, which is a cause of CI. However, another study showed that there was no correlation between β-blockers and depression the next year. A meta-analysis indicated β-blockers were related to the occurrence of vascular dementia (161, 162). Thus, further investigations are warranted to identify the specific relationship between β-blockers and CI.

##### Mineralocorticoid Receptor Antagonists

Mineralocorticoid receptors are ubiquitously expressed in limbic brain structures such as the hippocampus, amygdala, and prefrontal cortex. Findings showed that mineralocorticoid receptor antagonists (MRAs) decreased verbal learning, verbal memory, and visuospatial memory in adult population and impaired verbal memory and executive function in young depressed patients. However, MRAs improved verbal learning and visuospatial memory in elderly depressed patients. Thus far, the relevant research of MRAs and CI is relatively limited, and the impact of MRAs on CI is still controversial (163).

#### Effect of CI Medical Therapy on HF

Virtually all clinical trials of HF therapy have excluded patients with CI or dementia. There is limited data pertaining to the treatment for CI in HF. As a result, current guidelines are unable to provide evidence-based recommendations for diagnosis and treatment of patients with CI in routine clinical practice (164). Acetylcholinesterase inhibitors and memantine are considered as the first-line therapy for CI due to the benefit of concentration and memory. A position paper from the Study Group on Heart and Brain Interaction of the Heart Failure Association mentioned the favorable side effect profile for CI and the potential cardiovascular adverse events. For acetylcholinesterase inhibitors co-treated with β-blockers, digoxin, amiodarone, and calcium channel blockers may increase the risk for syncope or heart block. Dizziness, hypertension, angina, bradycardia, and HF may be observed in patients treated with memantine (165, 166).

### Novel Therapeutic Targets

Angiotensin-converting enzyme 2 (ACE2), angiotensin-(1-7) [Ang-(1-7)], and Mas have been identified as a new component of RAS, which constitute ACE2-Ang-(1-7)-Mas axis (167). Recently, Ang-(1-7) has showed to reverse HF-related CI in animal experiment. Ang-(1-7) is mainly produced by the hydrolysis of Ang II and the ligand for the Mas receptor. MAS is highly expressed in the hippocampus, a brain region related to memory function. Thus, the activation of Mas by Ang-(1-7) may play a protective role in brain. Hay et al. found that following 3 weeks treatment with systemic Ang-(1-7), the HF mice novel object recognition discrimination ratios were significantly better than the performance of mice with HF treated with saline (168). Ang-(1-7) also improved spatial memory in mice with HF without effect on cardiac function. Besides, 3 weeks of Ang-(1–7) treatment in the mice with HF resulted in a significant increase in plasma IL-1a, G-CSF, IL-16, and sICAM, which have been shown a neuroprotection in animal models of head injury or brain ischemia (169). The latest study demonstrated a novel glycosylated Ang-(1-7) peptide, Ang-1-6-O-Ser-Glc-NH_2_ (PNA5), which has greater brain penetration compared with the native Ang-(1-7) peptide in HF mice model. Moreover, after treatment with subcutaneous injection 1.0/mg/kg for 3 weeks, PNA5 activation of the Mas receptor reversed object recognition impairment in mice with HF and rescued spatial memory impairment. PNA5 treatment also decreased circulating pro-inflammatory cytokine, such as TNF- α, IL-7, and granulocyte cell-stimulating factor serum levels, while increasing that of the anti-inflammatory cytokine IL-10.

As an important inflammatory factor mediating the occurrence of CI in HF, TNF- α is also a potential therapeutic target. TNF-α inhibitor Etanercept improve cognitive function by increasing the density of dendritic spines in frontal and parietal cortex (76). In addition, Lidington et al. found that a TNF-α negative regulator, cystic fibrosis transmembrane conductance regulator (CFTR) may be a novel therapeutics target for CI in HF. By emulating the key features of HF-related CI, including reduced CBF and compromised neurologic function in mouse models, CFTR corrector compounds (C18) normalize pathological alterations in cerebral artery CFTR expression, vascular reactivity, and cerebral perfusion without affecting systemic hemodynamic parameters (170). Therefore, CFTR therapeutics may be a novel target to manage CI in HF.

### Non-pharmacologic Approaches for CI in HF

#### Life-Style Modification Physical Activity and Exercise

Studies indicated that the modification of lifestyle, including exercise and diet, can potentially improve cognition in patients with HF. Higher daily steps per day predicted better cognitive function and greater subcortical volume, with specific effects for the thalamus and ventral diencephalon (171). Redwine et al. also showed a greater MoCA score increases in patients with HF with Tai Chi or resistance band exercise compared to treatment as usual. Recently, Vellone et al. found that worse CI was independently associated with lower 6MWT scores. Of note, exercise capacity was associated with various cognitive domain, including visuospatial/executive, naming, attention, language, and orientation (19). Diet modification, such as low-salt diet, Mediterranean diet, and high-fiber diet, is also a feasible approach to preserving cognitive function and reducing risk of dementia. Several observational studies reported a protective association between certain nutrients (e.g., folate, flavonoids, vitamin D, and certain lipids) or food groups (e.g., seafood, vegetables, and fruit) and cognitive outcomes in older adults. A large randomized controlled trial further demonstrated a 2-year multidomain intervention of diet, exercise, cognitive training, and vascular risk monitoring could improve or maintain cognitive function in at-risk elderly people from the general population (172).

#### Device Therapy

Device therapy of HF has been reported contribute to cognitive improvement by improving cardiac output. Patients with moderate to severe HF enhanced cognitive outcomes within 3 months of cardiac resynchronization therapy (CRT) due to the improved left ventricular ejection fraction (LVEF) in response to CRT (173). Patients with improved LVEF showed better outcomes on measures of executive functioning, global cognition, and visuospatial functioning. Similarly, Zimpfer et al. indicated that successful ventricular assist devices implantation contributes to cognitive improvement by increasing cerebral blood flow in patients with advanced HF (174). Besides, as a common comorbidity of HF, AF exacerbates cognitive dysfunction and cerebral perfusion in patients with HF (94). Recently, a prospective case-control study assessed changes in cognitive function in 308 patients treated with AF catheter ablation and 50 medically managed controls, finding a significant improvement in cognitive function at 3 months and 1 year after ablation but not in the control group (175). This result supported that ablation may facilitate cognitive recovery from cerebral hypoperfusion by restoring sinus rhythm.

## Knowledge Gaps and Areas for Future Research

The prevalent CI in patients with HF bring a greater burden to the poor outcome and worsening quality of life. Even though great efforts sought to characterize the detrimental issue the with increasing awareness and advancement of the diagnostic tools, knowledge gaps still exist in this field. First, more prospective data on the role of HF in cognitive decline await future detailed investigations. Specifically, well-designed longitudinal studies with longer follow-up are warranted to illustrate the time cure in regard of: (1) the precise progression of cognitive decline from normal to dementia in patients with HF; and (2) the deleterious effect of varying degree of CI on survivor time and clinical course of HF. Additionally, comprehensive evaluations, including treatment effectiveness, compliance, caregiver burden and frailty, should be considered. Second, further research deserves to expound the contributory mechanisms involved in the pathophysiology of CI in HF to deepen understanding of heart-brain interaction. Third, systematic assessment of CI in HF with multiple imaging and neuropsychiatric approach, risk stratifications with specific biomarker profiles, and prediction models are expected to provide more prognostic information and guide therapy decisions. Fourth, despite the growing knowledge about the mutual malignant impact of HF and CI, little is known on promising targets for novel therapeutic interventions. In the future, research priorities outlined above will attract far more attentions in HF-CI interconnectivity. The availability of novel techniques, emerging and existing (repurposed) therapies, computational modeling and novel insights will help address these knowledge gaps.

## Conclusions

Heart failure (HF) and CI are increasing in prevalence and, when present together, are associated with significant mortality and morbidity. The underlying mechanisms of the link between cardiac dysfunction and brain pathologies in HF condition are still largely elusive. While patients with HF with dementia have great difficulties in daily life and are heavily dependent on caregivers, patients with CI are more independent. Routine screening of CI is needed in HF, even from using a simple tool like MoCA, thereby improving the efficiency of HF management. In patients with HF and CI, lifestyle change and risk factor control, standard HF therapy, and appropriate medication for CI should be standard of management. Further studies are needed not only to unravel the bidirectional pathology of the heart and cognition, but also to provide more efficient interventions in the brain following HF-associated conditions. Close collaboration between the HF and neurology specialists is essential in the early recognition and appropriate management of these patients. Future research to develop a consensus management guideline for patients with HF and CI, which is not yet available, is warranted.

## Author Contributions

MYang and DS searched and selected the references and wrote the first draft of the review. YW and MYan contributed toward literature review and interpretation of the manuscript. JZ and JR helped to determine the content and structure of the review and contributed to the writing and revision of the manuscript. All authors approved the final version of the paper.

## Funding

This study was supported by the National Natural Science Foundation of China (81770359 to JR), Beijing Health Technologies Promotion Program (BHTPP202004 to JR), Open Project Fund of State Key Laboratory of Molecular Developmental Biology of China (2021-MDB-KF-18 to JR), and Elite Medical Professionals Project of China-Japan Friendship Hospital (ZRJY2021-BJ01 to JR).

## Conflict of Interest

The authors declare that the research was conducted in the absence of any commercial or financial relationships that could be construed as a potential conflict of interest.

## Publisher's Note

All claims expressed in this article are solely those of the authors and do not necessarily represent those of their affiliated organizations, or those of the publisher, the editors and the reviewers. Any product that may be evaluated in this article, or claim that may be made by its manufacturer, is not guaranteed or endorsed by the publisher.

## Glossary

AD: Alzheimer's disease
Aβ: amyloid-β
ATP: adenosine triphosphate
ACEI: angiotensin converting enzyme inhibitor
ACE2: angiotensin-converting enzyme 2
AF: atrial fibrillation
Ang-II: angiotensin II
Ang-(1-7): angiotensin-(1-7)
APP: amyloid precursor protein
ARB: angiotensin II receptor blocker
ARNI: angiotensin receptor neprilysin inhibitor
BBB: blood-brain barrier
BMI: body mass index
BNP: brain natriuretic peptide
BP: blood pressure
CAMCOG: Cambridge cognitive examination
CBF: cerebral blood flow
CFTR: cystic fibrosis transmembrane conductance regulator
CI: cognitive impairment
CO: cardiac out
CRT: cardiac resynchronization therapy
CSF: cerebrospinal fluid
DM: diabetes mellitus
DPP4: dipeptidyl peptidase-4
DTI: diffusion tensor imaging
FAQ: functional activities questionnaire
fMRI: functional magnetic resonance imaging
GMD: gray matter density
HF: heartfailure
HFpEF: heart failure with preserved ejection fraction
HFrEF: heart failure with reduced ejection fraction
HIF-1: hypoxia inducible factor-1
IL: interleukin
LVEF: left ventricular ejection fraction
MCI: mild cognitive impairment
MMSE: mini-mental state examination
MoCA: Montreal cognitive assessment
MRI: magnetic resonance imaging
MTA: medial temporal lobe atrophy
NIRS: near-infrared spectroscopy
NPI-Q: neuropsychiatric inventory questionnaire
NT-proBNP: N-terminal pro-brain natriuretic peptide
p-GSK3β: phosphorylated glycogen synthase kinase 3β
PNA5: ang-1-6-O-Ser-Glc-NH2
PVM: perivascular macrophage
QoL: quality of life
RAS: renin angiotensin system
RCS: rapid cognitive screen
SGLT2: sodium glucose co-transporter 2
SLUMS: Saint Louis University mental status
ROS: subsequent reactive oxygen species
SPECT: single photon emission computed tomography
TLR-4: toll-like receptor-4
TNF-α: tumor necrosis factor-α
TSPO: translocator protein
VEGF-1: vascular endothelial growth factor-1
WMH: white matter hyperintensities
WML: white matter lesions
BACE: β-site amyloid precursor protein-cleaving enzyme-1
6MWT: 6-minute walk test.
